# Reducing prescribing of antibiotics for acute respiratory infections using a frontline nurse-led EHR-Integrated clinical decision support tool: protocol for a stepped wedge randomized control trial

**DOI:** 10.1186/s12911-023-02368-0

**Published:** 2023-11-14

**Authors:** Elizabeth R. Stevens, Ruth Agbakoba, Devin M. Mann, Rachel Hess, Safiya I. Richardson, Thomas McGinn, Paul D. Smith, Wendy Halm, Marlon P. Mundt, Katherine L. Dauber-Decker, Simon A. Jones, Dawn M. Feldthouse, Eun Ji Kim, David A. Feldstein

**Affiliations:** 1 NYU Grossman School of Medicine, New York, NY USA; 2 University of Utah Health, Salt Lake City, UT USA; 3 Baylor College of Medicine, Houston, TX USA; 4 University of Wisconsin School of Medicine and Public Health, Madison, WI USA; 5 University of Wisconsin School of Nursing, Madison, WI USA; 6 Feinstein Institutes for Medical Research, Northwell Health, Manhasset, NY USA; 7 Department of Medicine, Zucker School of Medicine at Hofstra/Northwell, Hempstead, NY USA

**Keywords:** Integrated clinical prediction rules, EHR, Implementation, Acute respiratory infections, Antibiotics, RCT

## Abstract

**Background:**

Overprescribing of antibiotics for acute respiratory infections (ARIs) remains a major issue in outpatient settings. Use of clinical prediction rules (CPRs) can reduce inappropriate antibiotic prescribing but they remain underutilized by physicians and advanced practice providers. A registered nurse (RN)-led model of an electronic health record-integrated CPR (iCPR) for low-acuity ARIs may be an effective alternative to address the barriers to a physician-driven model.

**Methods:**

Following qualitative usability testing, we will conduct a stepped-wedge practice-level cluster randomized controlled trial (RCT) examining the effect of iCPR-guided RN care for low acuity patients with ARI. The primary hypothesis to be tested is: Implementation of RN-led iCPR tools will reduce antibiotic prescribing across diverse primary care settings. Specifically, this study aims to: (1) determine the impact of iCPRs on rapid strep test and chest x-ray ordering and antibiotic prescribing rates when used by RNs; (2) examine resource use patterns and cost-effectiveness of RN visits across diverse clinical settings; (3) determine the impact of iCPR-guided care on patient satisfaction; and (4) ascertain the effect of the intervention on RN and physician burnout.

**Discussion:**

This study represents an innovative approach to using an iCPR model led by RNs and specifically designed to address inappropriate antibiotic prescribing. This study has the potential to provide guidance on the effectiveness of delegating care of low-acuity patients with ARIs to RNs to increase use of iCPRs and reduce antibiotic overprescribing for ARIs in outpatient settings.

**Trial registration:**

ClinicalTrials.gov Identifier: NCT04255303, Registered February 5 2020, https://clinicaltrials.gov/ct2/show/NCT04255303.

**Supplementary Information:**

The online version contains supplementary material available at 10.1186/s12911-023-02368-0.

**Table:** 

Contributions to the literature	
• Use of integrated clinical prediction rules (iCPRs) can reduce inappropriate antibiotic prescribing but they remain underutilized by physicians.	
• Given significant limitations to using a physician-driven iCPR model, determining whether the iCPR tasks can be delegated to other qualified members of the medical team is a key next step in the implementation process.	
• Implementation of a nurse-led model of iCPR for low-acuity ARIs may be an effective alternative to the physician-driven model.	
• This is the first protocol to study the use of a nurse-led model of iCPR implementation for antibiotic stewardship.	

## Background

Each year in the United States, 47 million unnecessary antibiotic prescriptions are provided to patients [1]. An estimated 80–90% of antibiotic prescribing occurs in outpatient settings such as doctors’ offices, urgent care facilities, and emergency departments [2–4]. In the United States an estimated 50% of all outpatient antibiotic prescriptions for ARIs are inappropriate [5, 6]. From 2011 to 2016, 62% of adult primary care patients with a diagnosis of acute bronchitis received antibiotics contrary to guideline recommendations against antibiotic treatment [7]. Patients with sore throats received antibiotics 61% of the time, while the prevalence of Group A streptococcus is only 10% in adults [8]. Unnecessary antibiotic prescriptions lead to a rise in antibiotic resistance, a major public health risk with 35,000 deaths each year due to antibiotic resistant bacterial infections [9, 10]. Most importantly, overprescribing of antibiotics for acute respiratory infections (ARIs) remains the most significant factor causing antibiotic resistance [11, 12].

There is a growing focus on promoting antibiotic stewardship both globally and within the United States [13, 14]. Appropriate antibiotic use and prescribing helps to ensure that lifesaving drugs are available for patients when they really need them, reduce unnecessary adverse drug events and associated healthcare costs. Despite efforts to educate patients and clinicians regarding the ineffectiveness of antibiotics in most ARIs, including the Center for Disease Control and Prevention’s (CDC’s) “Get Smart” campaign, recently rebranded “Be Antibiotics Aware,” since 2003, [15, 16] prescribing rates have remained relatively stable since the late 1990’s and use of broad spectrum antibiotics has increased [17]. The factors underpinning the persistent inappropriate prescribing of antibiotics for ARIs are varied and include diagnostic uncertainty, time pressures, cognitive fatigue, fear of serious complications, and the need to meet patient expectations, which includes a perceived patient demand for antibiotics [18–25]. Prescriber-level barriers to antibiotic stewardship can potentially be addressed through clinical decision support (CDS) interventions to decrease inappropriate prescribing of antibiotics [26–29].

Clinical prediction rules (CPRs) are a form of CDS that function to provide real-time evidence-based data to assist clinicians (physicians, nurse practitioners and physician assistants) with decision making. CPRs can be integrated into the electronic health record (EHR) to bring best practice guidelines to the point of care [26]. Indeed, CPRs have already been validated to accurately distinguish between viral and bacterial respiratory infections [30–32]. However, there is an ongoing need to develop implementation strategies to ensure the uptake and appropriate use of these interventions.

In a previous study (iCPR1), our team developed and tested two EHR-integrated CPRs (iCPRs) to reduce antibiotic prescribing for ARIs in primary care practices [26]. By providing an estimate of the likelihood of a patient having either pneumococcal pneumonia or group A streptococcus, the iCPRs helped clinicians determine a patient’s risk for bacterial infection and provided order sets tailored to patient risk. While the iCPRs were available, clinicians ordered significantly fewer diagnostic tests (e.g., strep test, chest x-ray) and antibiotics (a 35% reduction) [26]. However, when assessing the generalizability of these findings in primary care practices across two academic medical institutions (iCPR2), the iCPRs showed no significant impact on diagnostic testing or antibiotic prescribing rates [33]. In particular, there were low adoption rates of the iCPR tools indicating potential implementation barriers to antibiotic stewardship iCPRs among physicians.

Due to the limitations associated with a physician-driven iCPR model, such as ‘alert fatigue’ and time constraints, [34, 35] determining whether the iCPR tasks can be delegated to other qualified members of the medical team is a key next step in the implementation process. Registered nurses (RN) have been used to effectively improve ambulatory care across a number of chronic diseases, [36] and for the treatment of acute minor illness, have achieved equivalent symptom resolution as compared to physicians [37, 38]. Therefore, implementation of an RN-led model of iCPR for low-acuity ARIs may be an effective alternative to the physician-driven model.

## Methods

After refinement of CDS tools using qualitative usability testing, we will conduct a stepped-wedge practice-level cluster randomized controlled trial (RCT) examining the effect of iCPR-guided RN care for low acuity patients with ARIs. Specifically, this study aims to: (1) determine the impact of iCPRs on diagnostic test ordering and antibiotic prescribing rates when used by RNs; (2) examine resource use patterns and cost-effectiveness of RN visits across diverse clinical settings; (3) determine the impact of iCPR-guided care on patient satisfaction; and (4) ascertain the effect of the intervention on RN and physician burnout. All study methods have been approved by the NYU Langone Health Institutional Review Board (IRB) (NYULH Study Number: i19-01222).

The primary hypothesis to be tested is: Implementation of RN-led iCPR tools will reduce antibiotic prescribing for ARI across diverse primary care settings.

### Setting

This is a multi-site study being conducted at 48 primary and urgent care practices associated with New York University Langone Health (NYULH), Northwell Health, the University of Wisconsin Hospitals and Clinics and University of Utah Health. NYULH, Wisconsin, and Utah use the Epic EHR system (Epic Systems, Verona, WI), while Northwell Health uses the Allscripts TouchWorks EHR system (Altera Digital Health, Niagara Falls, NY).

#### Eligibility criteria

Eligible practices are general internal medicine (GIM), family medicine primary care (FM), and urgent care clinics. Each practice must have at least one RN full time equivalent capable of performing triage within the EHR and conducting RN on-site visits. Patients with a primary complaint of cough or sore throat, who meet the low-acuity criteria, will be included in the analysis. Low acuity patients have symptoms that do not require urgent evaluation and have limited comorbidities. See Table 1 for the triage protocol that will be used to identify patients appropriate for an RN visit. Triage will occur over the phone in GIM and FM practices and in person in urgent care practices. Eligible patients at FM clinics will include those ages 3–70 for sore throat and 18–70 for cough. GIM and urgent care clinics will include patients 18–70 for both sore throat and cough.

**Table 1:** Implementation Outcome measures

Outcome	Measure (data source)	Frequency
User-centered adaptation	Stakeholder interviews and workflow analysis	Pre-implementation
Workflow integration	Usability testing and piloting	Pre-implementation
Acceptability • Perceived usefulness • Perceived ease of use	TAM3 questionnaire (RN survey) Live usability testing	6-months 6-months
Fidelity	Live usability checklist	6-months
Adoption	# RN triage completed (EHR) # risk calculators completed (EHR) # order sets completed (EHR) # RN visits (EHR)	6-, 12-months
Appropriateness	Satisfaction (RN, clinician, patient survey)	Pre-implementation, 6-months
Sustainability	Factors re: maintenance and scaling of RN iCPR (interviews)	12-months
Impact	ARI encounters with inappropriate antibiotic Rx (%) (EHR) Diagnostic testing (Rapid strep, CXR) (EHR)	Post-intervention
Cost	Cost-effectiveness (EHR, reported training times, institutional salaries)	Post-intervention

#### Recruitment

Institutional physician and nurse leadership is engaged and supportive of the study. Presentations to physicians and practice managers will be used to obtain initial buy-in. Eligible practices will then be recruited from a list of affiliated outpatient practices. Study site leads will approach practice managers and medical directors at each practice to explain the study and ask for study participation. All study procedures will be performed in accordance with the Declaration of Helsinki. This research protocol was reviewed and approved by the NYU School of Medicine and NYU Langone Health Institutional Review Board (NYULH Study Number: i19-01222). Informed consent will be obtained from all the participants and/or their legal guardians. For patients, the NYU Langone Health IRB waived the informed consent. NYU served as the central IRB for this study.

### Usability testing

Think aloud testing [39] will be performed sequentially to evaluate the usability of each of the EHR tools including triage and RN visit formatted notes, CPRs, and order sets. Each institution will conduct a group think aloud session with 3–4 RNs. We will purposefully sample RNs from different practices including FM, GIM and urgent care. The sessions are performed via videoconference and include a moderated individual use of the tools in mock clinical cases followed by debrief questions in a focus group setting. Sessions are recorded and coded by a single coder per institution. Usability sessions will continue until data saturation is reached. Results are analyzed prior to the next testing session to enable rapid tool iteration. The usability testing may result in major changes such as in the order and wording of the triage notes, wording of the RN visit physical exam and antibiotic directions in the order sets.

### Trial design

**Fig. 1:**
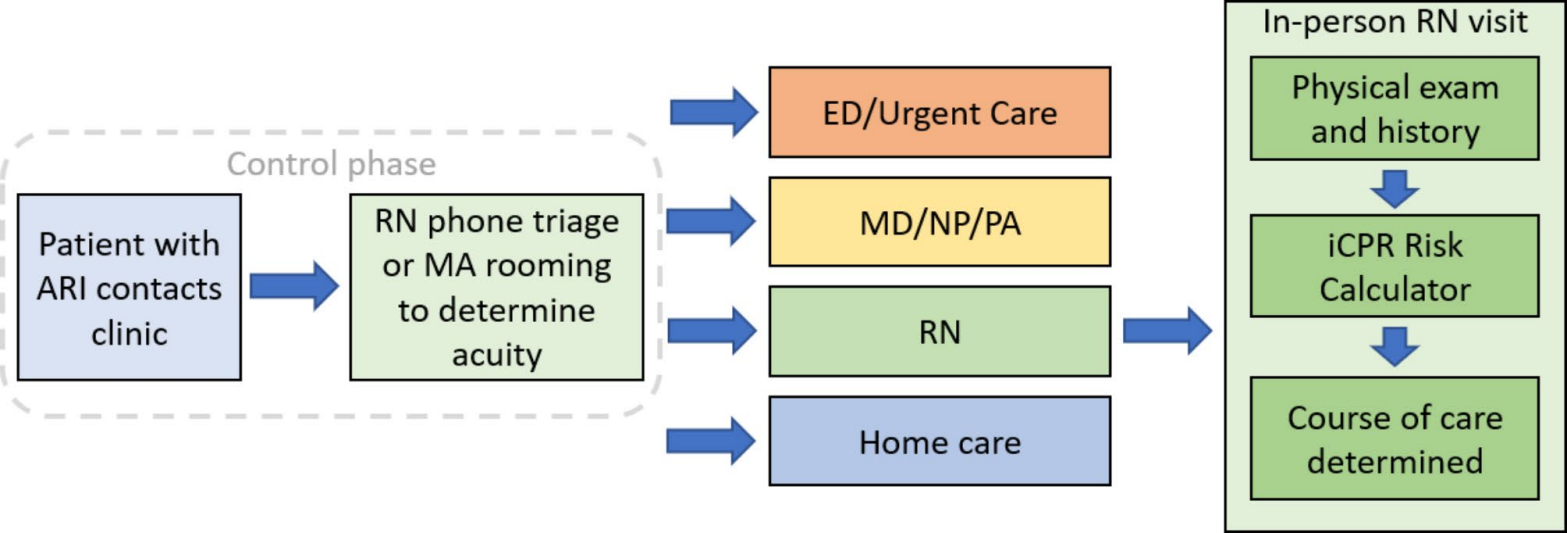
Intervention workflow. Note: ARI-acute respiratory infection; RN-registered nurse; MA-Medical assistant; ED-emergency department; MD-medical doctor; NP-nurse practitioner; PA-physician’s assistant

**Fig. 2:**
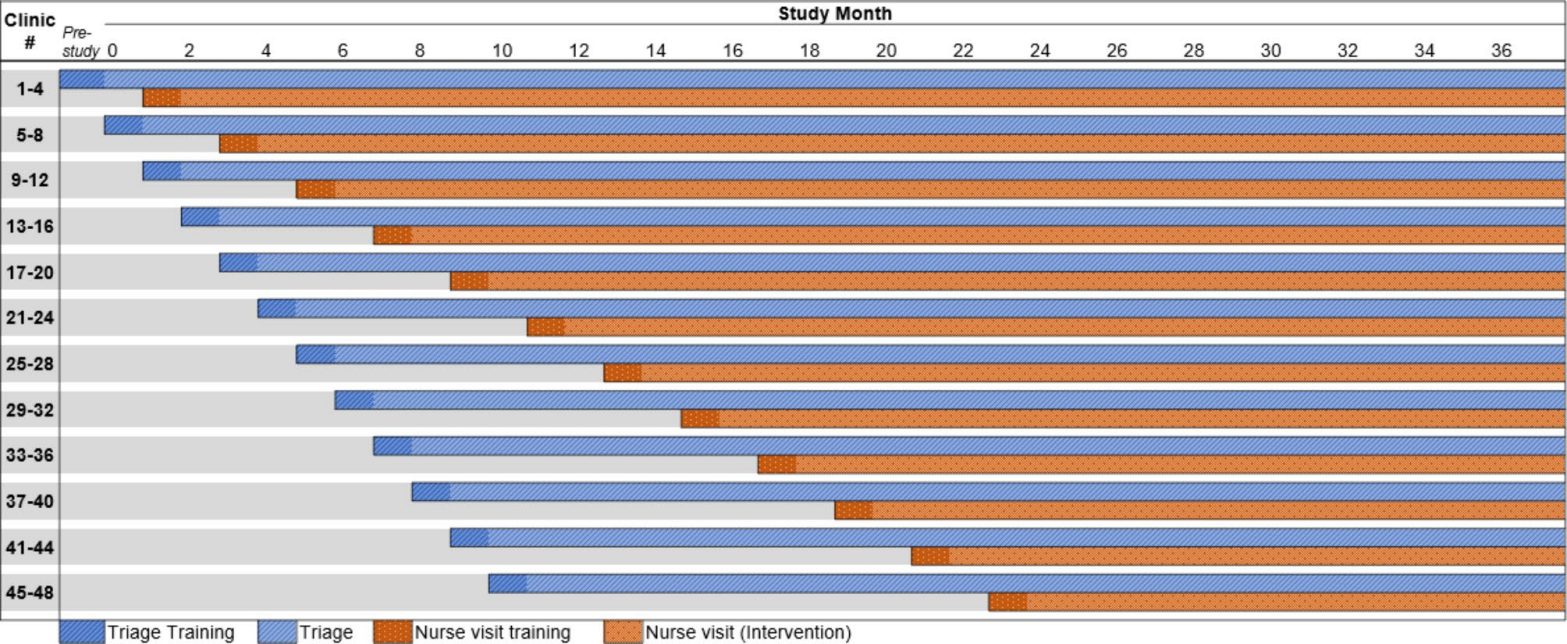
Step-wedge implementation of triage and RN visit study components

We will conduct a stepped-wedge practice-level cluster randomized control trial with 48 practices with four each step randomized over 24 months. The study control phase consists of documented triage of cough and sore throat patients including an assessment of symptom acuity and eligibility for a RN visit, followed by an intervention phase consisting of triage in addition to an in-person iCPR-guided RN visit for patients with low-acuity ARIs (Fig. 1). Each practice will serve as its own control prior to intervention implementation. Each practice will start in the control phase and practices will ‘step-in’ to the intervention phase at two-month intervals (Fig. 2). The timing of the steps will ensure each practice has at least two months of control period data and at least 12–36 months of exposure to the intervention.

#### Randomization

Computer-generated randomization will be used to determine clinic step group. To ensure a more even distribution of practice characteristics across steps, randomization will be stratified by covariates including medical center affiliation, practice type, and volume of ARI visits. Covariate-constrained randomization is preferred to simple randomization in this setting because 48 practices is unlikely to be a large enough number to ensure that a simple random assignment will balance the distribution of important confounders across steps [40].

Due to COVID-19 pandemic staffing shortages, some practices would be unable to implement the RN visits after initial enrollment. To be pragmatic and allow these otherwise eligible practices to participate in the study, we will perform randomization in two separate groups: An early group prepared to start RN visits in the first 12 months of the study, and a late group that will be randomized to start RN visits in months 13–24.

### Outcomes

The main study outcome is the proportion of ARI encounters with an antibiotic prescription. Secondary outcomes include: proportion of ARI encounters with an appropriate antibiotic prescription in the control and intervention steps (see supplement), as defined by Meeker et al., [41] the proportion of patients receiving testing, patient satisfaction, RN burnout, physician burnout and measures of implementation. The proportion of patients receiving testing is the number of patients who have a rapid strep or chest x-ray ordered with a denominator of patients that triage to a nurse visit. Patient satisfaction will be measured using the seven item Short Assessment of Patient Satisfaction (SAPS) scale that was developed to assess patient satisfaction with their treatment [42]. All patients that triage as appropriate for an RN visit will receive the survey via their patient portal. All RNs and physicians at participating clinics will receive the 10 item Mini Z survey that measures work stress and burnout [43]. Evaluation for potential harms of RN visits will be evaluated in patient practice, urgent care or ED visits or hospitalizations, and new antibiotic prescriptions within two weeks of their sentinel practice visit.

#### Implementation outcomes

Implementation outcome measures are presented in Table 2. We will use the Technology Acceptance Model (TAM3) [44] to guide implementation and assess technology-based implementation outcomes. TAM3 asserts that perceptions of usefulness and ease-of-use by end-users will directly influence intention to use a new technology, leading in turn to its adoption. Participating RNs will be surveyed after they start RN visits to determine their perceptions of the usefulness and ease of use of the EHR tools (Table 2). In addition to TAM3, we will use Proctor’s Implementation Outcomes Framework (IOF) [45] to guide our assessment of implementation outcomes. The IOF was proposed to establish a taxonomy for implementation outcomes and to standardize these outcomes for further research. Implementation findings will be used to [1] drive post-implementation tool optimization and [2] create an implementation toolkit and sustainability plan.

**Table 2:** Triage criteria

	Sore Throat		Cough	
**ED or Urgent Care**	Difficulty breathing		Frothy pink sputum	Continuous chest pain
	Inability to open mouth completely		Aspiration of a foreign body	Severe wheezing not relieved with inhaler
	Inability to swallow saliva		Hives	Blue lips
	Wheeze with inhaling		Chest pain with exertion	Feeling of suffocation
			Decreased level of consciousness	Inability to speak more than 2 words
			Severe shortness of breath	Inhalation of smoke or noxious fumes
**Clinician visit**	Sore throat for > 1 week without improvement		Previous visit for the same episode	Inability to breathe while lying down
	Symptoms of dehydration		Coughing up blood	Sudden weight gain
	Productive cough		Grossly bloody sputum	New leg or ankle swelling
	Temperature > 102 °F		Cough for > 2 weeks without improvement	Coughing or choking after eating
			Symptoms of dehydration	Symptoms requiring a visit and special population
**RN visit**	Sore throat for greater than 24 h	Difficulty swallowing due to pain	Productive cough without similar nasal discharge	Chest pain with coughing or deep breath
	Fever < 102 °F	Swollen lymph nodes	Blood streaked sputum	Severe cough
	Patient requests visit	Spots on back throat	Shortness of breath	Patient requests visit
	Close contact with strep pharyngitis		Inability to sleep due to coughing	
**Home care**	No symptoms requiring a visit		No symptoms requiring a visit	
* Special populations (Clinician visit if visit is required)	Patient < 18 y/o Patient > 70 y/o Currently pregnant or may be pregnant Immunocompromised End Stage Renal Disease on dialysis Chronic Kidney Disease Stage 4 Completed antibiotics within the past 2 weeks Previous strep testing during same illness		Patient < 18 y/o Patient > 70 y/o History of blood clots History of Asthma History of chronic lung disease History of congestive heart failure	Currently immunosuppressed Being treated for cancer Completed antibiotics within the past 2 weeks Chest trauma in the past 2 weeks

#### Data Collection

Clinical data will be collected via each institution’s EHR database. Discrete data elements built into the triage tool will be used to collect triage outcome and patient appropriateness for a RN visit. Clinical encounter outcomes will be collected for all patients with a clinician or RN visit with a reported ARI diagnostic code. Surveys will be sent to patients two weeks after an ARI visit. RNs and clinicians will complete surveys at baseline, six months, and 12 months after implementation. Semi-structured interviews will be conducted with medical directors, RN managers, and other key stakeholders.

### Statistical analysis

We will estimate the proportion of ARI encounters with antibiotic prescribing in the intervention and control steps to calculate 95% CIs while adjusting for clustering by site and time trend [46]. The odds ratio and confidence intervals of the proportion of ARI visits with antibiotic prescriptions during the control vs. intervention phase will be calculated using nonlinear mixed model effects using the R software’s geePack to account for cluster, time and intervention and repeated measures over time implementing the approach of Hussey & Hughes [47]. Generalized estimating equations (GEE) will be used as we have a binary response and substantial variability of cluster sizes (coefficient of variation = 0.62). The same approach will be used to compare rates of diagnostic testing (rapid strep antigen or chest x-ray) and additional visits in the intervention and control steps. Using similar methods, we will examine sex differences in main outcomes using sex as a biological variable. We calculated the sample sizes needed to test our primary hypothesis that iCPRs lead to a lower rate of antibiotic prescribing per eligible ARI encounter at the end of the study period (36 months). Based on data from our previous RCT we found an intraclass correlation coefficient (ICC) of 0.08 and average number of encounters per cluster per year of 800. Effect sizes were based on published results [33]. The power calculation was to find a power of 90% with an alpha of 0.05 and informed our plan to implement at 48 and up to 60 clinics over 24 months with 2 clinics per month being added to the intervention.

#### Cost-effectiveness

Cost-effectiveness analyses will be conducted from both a health care system and societal perspective. Cost-effectiveness will be reported in terms of the incremental cost-effectiveness ratio (ICER) [48] as the difference in costs between the Intervention and Control steps of the intervention divided by the difference between the steps in antibiotic prescribing. Costs are calculated by category (e.g., training and set-up costs, staff time and burden, administrative costs, patient costs and burden, health care costs borne by third-party payers or paid out-of-pocket by patients) as detailed in the Impact Inventory called for by the Second US Panel on Cost-Effectiveness in Health and Medicine [48]. Societal costs are more comprehensive than health care system costs in that patient costs are included in the analysis. We will have data on the number of patient contacts and patient visits associated with each ARI episode. Sensitivity analyses will be performed to introduce uncertainty about the rates of uptake of the intervention protocol and success in reducing antibiotic prescribing. Monte Carlo-based simulation estimation will use the rates of antibiotic prescribing observed as a reference to simulate a cohort of post-implementation participants and a cohort of usual care participants [49]. These probabilistic sensitivity analyses will estimate the elasticity of the cost per patient with ARI relative to differential changes in antibiotic prescribing.

### Intervention components

#### Control phase

Practices will remain in a control phase for at least two months prior to entering the intervention phase. During the control period, practice RNs will perform telephone triage for patients reporting cough and/or sore throat symptoms to assess acuity, specifically their risks of pneumonia and/or strep pharyngitis. This will guide the need for a clinician visit, urgent care or ED visit, and the appropriateness of an in-person iCPR-guided RN visit. In the case of urgent care visits, where a phone triage is not possible, medical assistants (MAs) will document patient symptoms during their rooming procedures for acuity assessment and determination of RN visit appropriateness. Patients with known COVID-19 infection will be excluded from RN visits.

After triage is completed, patients that triage to a RN visit will be seen as a usual care clinician visit. This control phase will serve to assess the volume of RN-visit eligible patients and the antibiotic prescribing practices under the standard of care. Current standard of care is that patients requiring an in-person visit are seen by a clinician who decides whether to do additional testing and prescribe antibiotics. Clinicians at the study sites are not provided with any tools to help determine patients risk or help with antibiotic choice.

The triage tool consists of a prepopulated note template integrated into the EHR system designed to document patient symptoms and severity, and determine level of triage, including appropriateness for an iCPR-guided RN visit. The triage tool varies slightly between health systems due to EHR system capabilities. The tool guides RNs to triage patients into one of four pathways based on symptom severity and medical history. Triage criteria can be found in Table 1 and the potential routes for the patient include: (1) must be seen immediately in an emergency department or urgent care setting; (2) requires a clinician visit; (3) low acuity and suitable to see a RN in the practice; (4) no visit required and patient receives educational material and home-care instructions. Triage algorithms were based on institutional triage resources for decisions about emergency department/urgent care visits, practice visits and home care [50]. Criteria to identify low acuity patients appropriate for RN visits were agreed upon by consensus of study clinicians across all institutions in addition to a review of the criteria by local clinical experts. Of note, patients with cardiac and pulmonary diseases, as well as advanced age and malignancy, are considered a special population and are not eligible for RN visits even with low acuity cough/sore throat symptoms. See Supplement Figure S1 for a triage tool example.

#### Intervention phase

After completion of the control phase, at their randomized assigned start times, practices will enter the intervention phase. The intervention phase includes continued use of the triage tool described in the control phase with the addition of an in-person iCPR-guided RN visit, which will replace standard of care for low-acuity patients.

##### RN visit

During the intervention phase, eligible patients triaged as low-acuity will be recommended for an in-person RN visit at their primary practice site that will replace the standard-of-care clinician visit. In primary care practices, RN visits will be scheduled within 36 hr of triage. During the RN visit, guided by iCPR tools, the RN will evaluate patients to determine their risk of bacterial infections of strep pharyngitis (sore throat) or pneumonia (cough). RNs will utilize prepopulated note template EHR tools to conduct a focused history and physical exam. If red flag signs of more severe illness are identified during the RN visit, the visit will be converted to a standard-of-care clinician visit. See Supplement Figure S2 for a RN-visit tool example.

##### iCPR tools

**Fig. 3:**
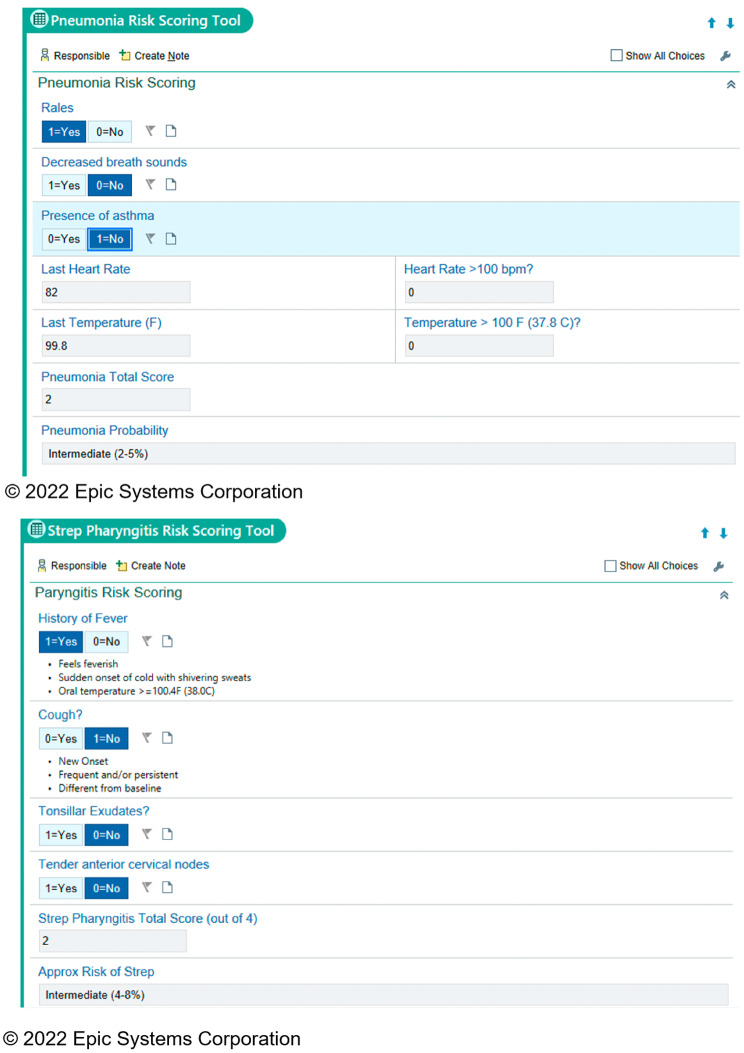
iCPR risk calculator tools for **(a)** pneumonia and **(b)** strep pharyngitis. Tools are copyrighted by Epic Systems Corporation, 2022

Once an RN has completed the patient history and physical exam, they will use an iCPR tool integrated into the EHR specific to cough or sore throat to calculate the risk of bacterial infection. The evidence-based iCPR tools are informed by the validated CPRs previously used in the iCPR1 and iCPR2 studies. The sore throat CPR is based on the Centor criteria for adults with sore throat, which include four components: absence of cough, pharyngeal exudates, tender anterior cervical lymphadenopathy, and fever [51]. The McIssac criteria used for children with sore throat mirror Centor criteria with the addition of patient age [32]. The cough CPR uses the Heckerling criteria for adults with risk of pneumonia which include five components: fever, increased heart rate, crackles, decreased breath sounds, and absence of asthma [31]. There are no known validated CPRs for children with cough. Upon completion of the iCPR risk calculator, the level of risk with an approximate probability of having either strep pharyngitis or pneumonia are displayed (Fig. 3).

##### Order sets

After completion of the iCPR risk calculator, the EHR will link the RN to an order set specific to the level of risk, age, and known allergies of the patient. An order set for patients at low risk of bacterial infection will contain patient instructions with criteria for when to call the practice and recommendations on symptomatic care such as over-the-counter cold medicine and analgesics. The order set for patients at intermediate or high risk for strep pharyngitis or pneumonia will recommend further diagnostic tests (rapid strep antigen or chest x-ray). If the test result is positive, then the order set will recommend antibiotics. These patients will also receive instructions on symptomatic care. Based on state RN scope of practice regulations, at some institutions a physician will be required to verify the RN’s physical exam findings, authorize any pending orders, and sign off on the assessment and treatment plan. Physician-time spent confirming visit details should be minimal. In this manner the iCPR tools will support the practice of evidence-based care and ultimately foster appropriate antibiotic prescribing.

### RN Training

RNs will receive extensive training prior to implementation. The training is divided into two components: triage and RN visit. Training will occur within two weeks before starting triage and starting RN visits, respectively. Training will be provided in a hybrid format. Triage training includes online materials plus virtual training. Triage training materials contain a project overview, triage criteria and review of triage tools. RN visit training includes four online, interactive modules and an in-person training session. The online modules are about 15 min each and contain lessons on taking a history, physical exam, and CPRs for determining patient risk as well as appropriate treatment (testing and/or antibiotics) based on risk. The one-hour in person training will take place at each practice individually and will focus on visit workflows and refreshing physical exam skills. At least two facilitators will lead the in-person session using a standardized facilitator guide to ensure that content is uniformly taught across sites. Live models will be used for physical exam skills training. To further standardization, all facilitators will attend a training session led by the study training developers to review training components.

## Discussion

Our study is a novel EHR-supported antibiotic stewardship implementation study. It is innovative because it: (1) leverages CDS with risk stratification to enable RNs to lead initial ARI assessment and treatment while practicing at the top of their license, (2) uses an evidence-based implementation framework to evaluate outcomes and identify barriers, facilitators and lessons learned, and (3) evaluates the cost of the intervention, which is critical for increasing dissemination potential. This is significant because it is expected to provide a scalable implementation model leveraging CDS and RN training tools to decrease antibiotic overuse, lower the cost of care, and ultimately reduce antibiotic resistance.

To our knowledge, this study represents the first implementation of a RN-led iCPR model specifically designed to address inappropriate antibiotic prescribing. This study will therefore fill a critical gap in the evidence and provide guidance on whether shifting care of low-acuity patients with ARIs to RNs can overcome barriers to the implementation of physician-facing iCPRs to reduce antibiotic overprescribing for ARIs in outpatient settings.

### Study limitations

Due to variability in health system characteristics, such as EHR type used, and state policies governing the scope of RNs’ practice, adjustments to the study protocol are likely to be needed on a site-to-site basis. Anticipated modifications include adaption of tool functionality to alternate EHR systems and restructuring of triage and RN visits to accommodate local regulations and practice structure. While potentially limiting the ability to compare outcomes between sites, these anticipated protocol differences make this study more generalizable for implementation in a variety of settings. In addition to protocol modifications required due to local circumstances, we are mindful that we must be pragmatic operating in the midst of COVID-19, as we have experienced early challenges with practice recruitment as each partner site has been impacted in unique ways through their institutional and state policies. Study teams at each site have been engaged in ongoing conversation with leadership, clinical, and administrative staff to iteratively build out the project workflow to maintain adherence to local COVID-19-related policies and prepare for future changes to policies. With the emergence of various strains of COVID-19, we have experienced that some clinical staff and clinicians are hesitant to have face-to-face visits with patients with ARI symptoms. In addition, the number of in-person ARI visits has also been impacted by the increased use of telemedicine visits.

## Conclusions

This study represents an innovative approach to using an RN-led iCPR model specifically designed to address inappropriate antibiotic prescribing. This study has the potential to provide guidance on the effectiveness of delegating care of low-acuity patients with ARIs to RNs to increase use of iCPRs and reduce antibiotic overprescribing for ARIs in outpatient settings.

### Electronic supplementary material

Below is the link to the electronic supplementary material.

Supplementary Material 1

## Data Availability

Not applicable.
