# Supplementary material for: Reducing prescribing of antibiotics for acute respiratory infections using a frontline nurse-led EHR-Integrated clinical decision support tool: protocol for a stepped wedge randomized control trial
Source: BMC Med Inform Decis Mak. 2023 Nov 14;23:260. doi: 10.1186/s12911-023-02368-0 (PMC10644670; doi:10.1186/s12911-023-02368-0)
Supplement: Supplementary file 1 — Supplementary Material 1 [file 12911_2023_2368_MOESM1_ESM.docx]

**Supplementary Materials**

**Exhibit S1. Methods for measurement of inappropriate antibiotic prescribing**

As per the methods set forth by Meeker et al (2016), antibiotic-inappropriate diagnoses will include any antibiotic prescription given nonspecific upper respiratory tract infections, acute bronchitis, and influenza. In a modification to Meeker, inappropriate diagnoses will also include acute pharyngitis without a positive rapid strep test or throat culture for group A streptococcus. Diagnosis codes for acute rhinosinusitis will be excluded from the inappropriate category because guidelines permit antibiotic prescription when certain criteria are met, and we will not have the data necessary to identify this antibiotic-appropriate subset. We will translate the Meeker et al. International Classification of Diseases, Ninth Revision (ICD-9) codes into ICD-10 codes for these analyses.

| **Antibiotic-inappropriate diagnoses ICD-9 code and their ICD-10 translations** | | | | | |
| --- | --- | --- | --- | --- | --- |
| **ICD-9** | **ICD-10** | **General Definition** | **ICD-9** | **ICD-10** | **General Definition** |
| 460 | J00 | Acute nasopharyngitis | 466.1  466.11  466.19 | J21  J21.9  J21.0  J21.1  J21.8  J21.9 | Acute bronchiolitis |
| 464  464.0  464.00  464.1  464.10 | J04  J04.0  J04.1  J04.10 | Acute laryngitis  Acute tracheitis |  |  |  |
| 464.2  464.20 | J04.2 | Acute laryngotracheitis | 487  487.1  487.8 | J09  J09.X  J09.X1  J09.X2  J09.X3  J09.X9  J10  J10.1  J10.8  J10.81  J10.82  J11.1  J11.2  J11.8  J11.81  J11.82  J11.83  J11.89 | Influenza |
| 464.4 | J05  J05.0 | Acute obstructive laryngitis |  |  |  |
| 464.50 | J04.3  J04.30 | Supraglottitis |  |  |  |
| 465 | J06 | Acute upper respiratory infections of multiple or unspecified sites |  |  |  |
| 465.0 | J06.0 | Acute laryngopharyngitis |  |  |  |
| 465.8  465.9 | J06.9 | Acute upper respiratory infection, unspecified |  |  |  |
| 466 | n/a | Acute bronchitis and bronchiolitis |  |  |  |
| 466.0 | J20  J20.4  J20.5  J20.7  J20.9 | Acute bronchitis |  |  |  |
| 490 | J40 | Bronchitis, not specified as acute or chronic |  |  |  |
| N/A ^a^ | J02^b^  J02.8^b^  J02.9^b^ | Acute pharyngitis |  | | |
| ^a^ Not in original Meeker method  ^b^ If not accompanied by positive rapid strep test or group A streptococcus throat culture | | | | | |

Meeker D, Linder JA, Fox CR, Friedberg MW, Persell SD, Goldstein NJ, et al. Effect of Behavioral Interventions on Inappropriate Antibiotic Prescribing Among Primary Care Practices: A Randomized Clinical Trial. JAMA. 2016;315(6):562-70.


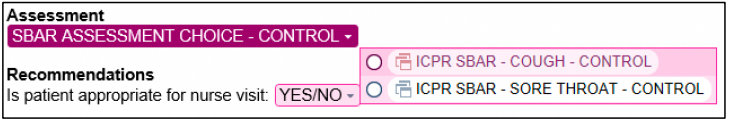
**
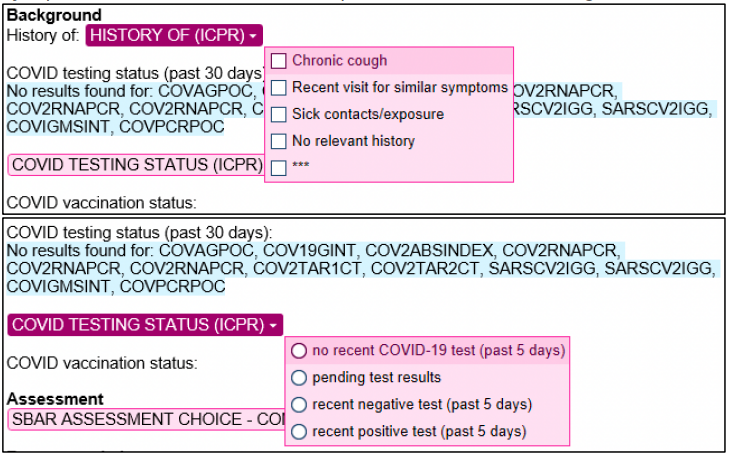

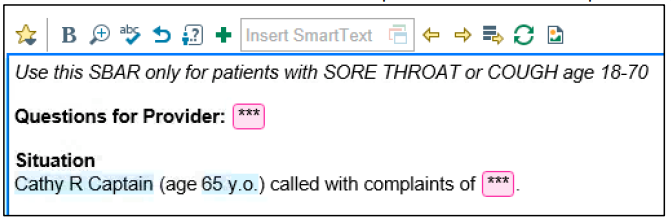
Supplement Figure S1**. Triage tool example


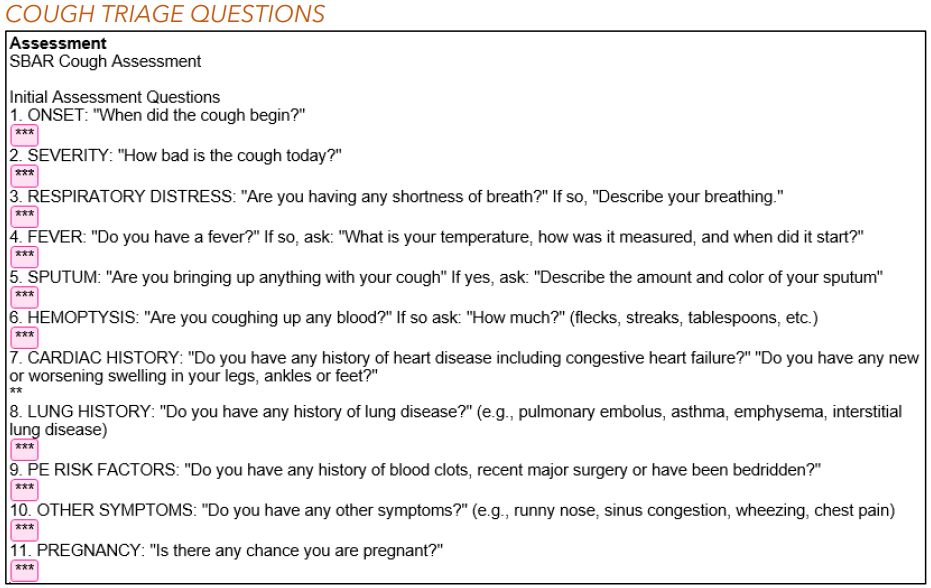


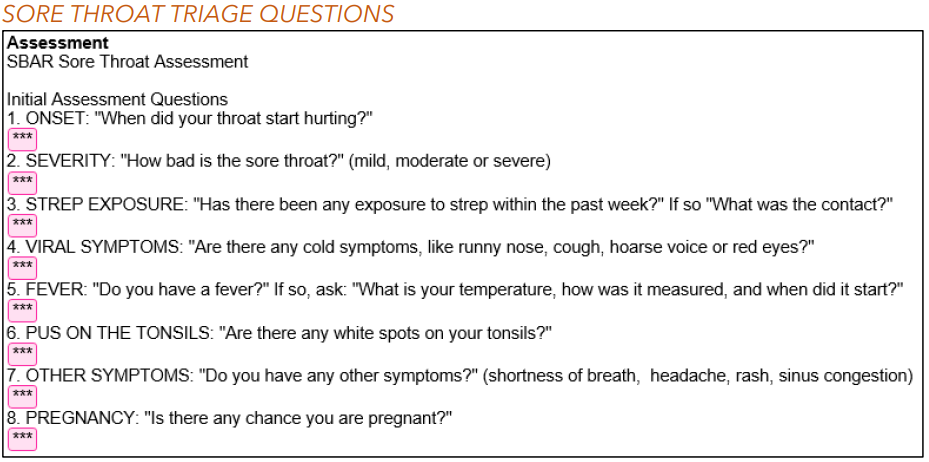


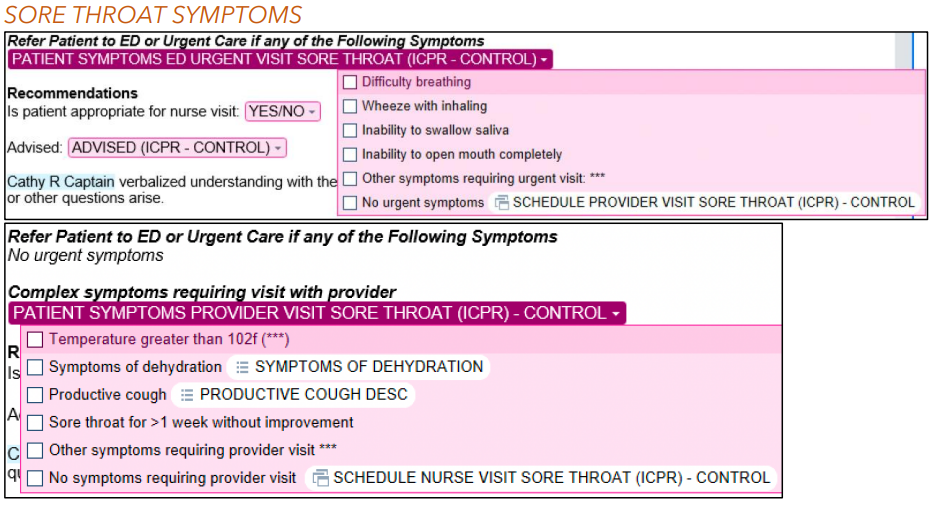

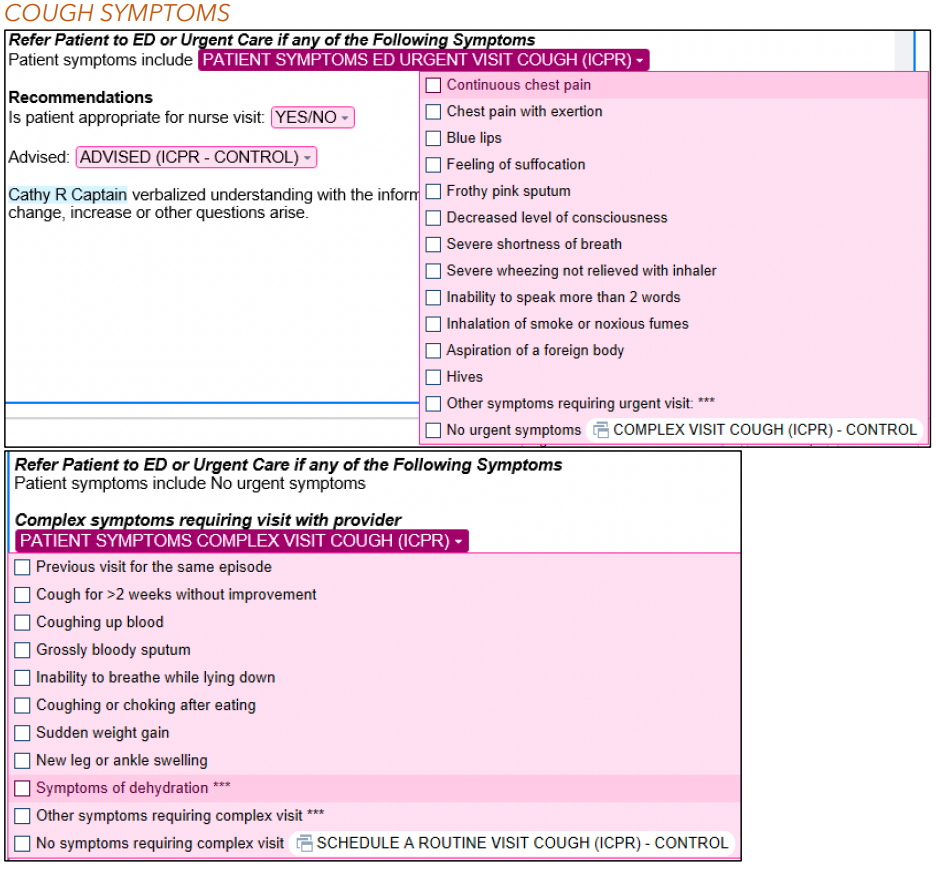


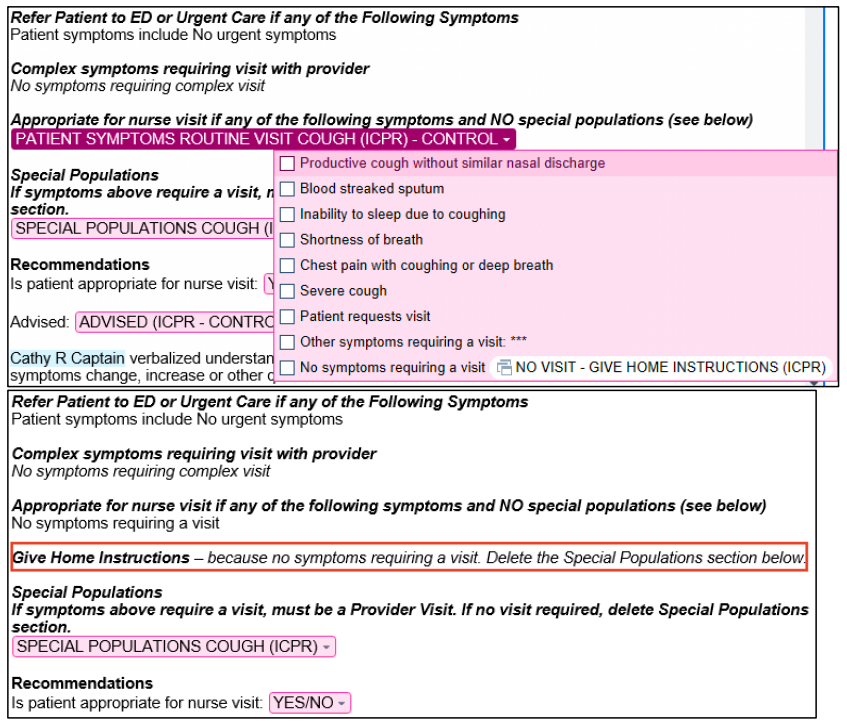

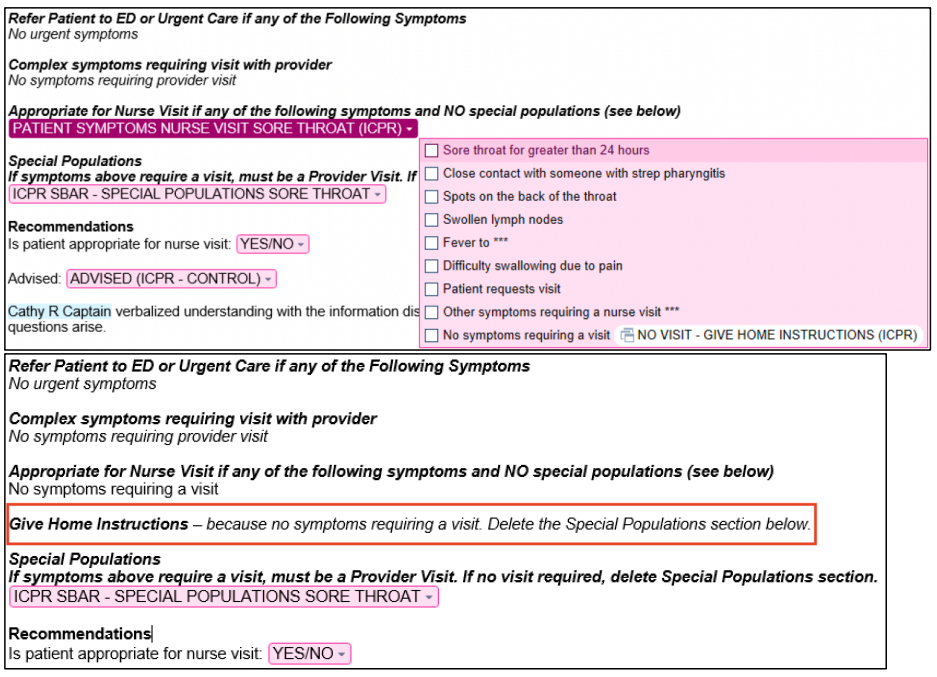


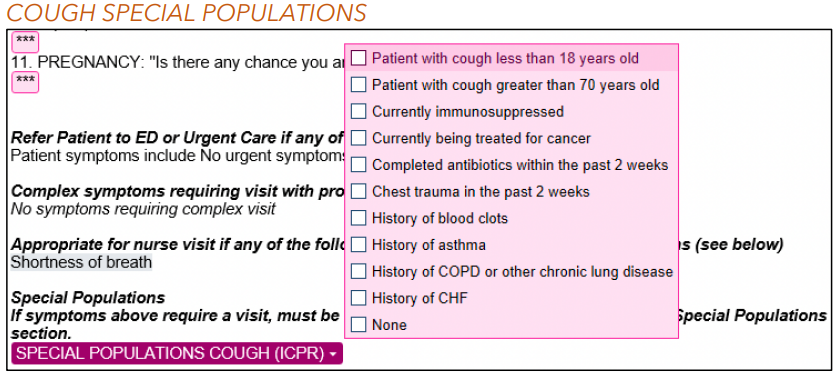


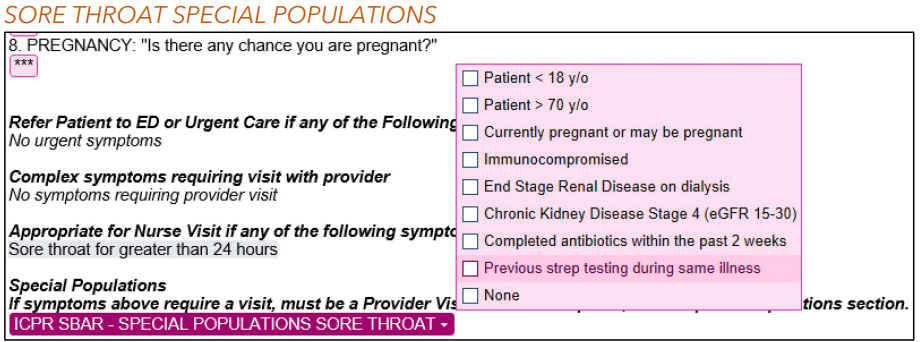


© 2022 Epic Systems Corporation

**Supplement Figure S2**. RN visit tool example


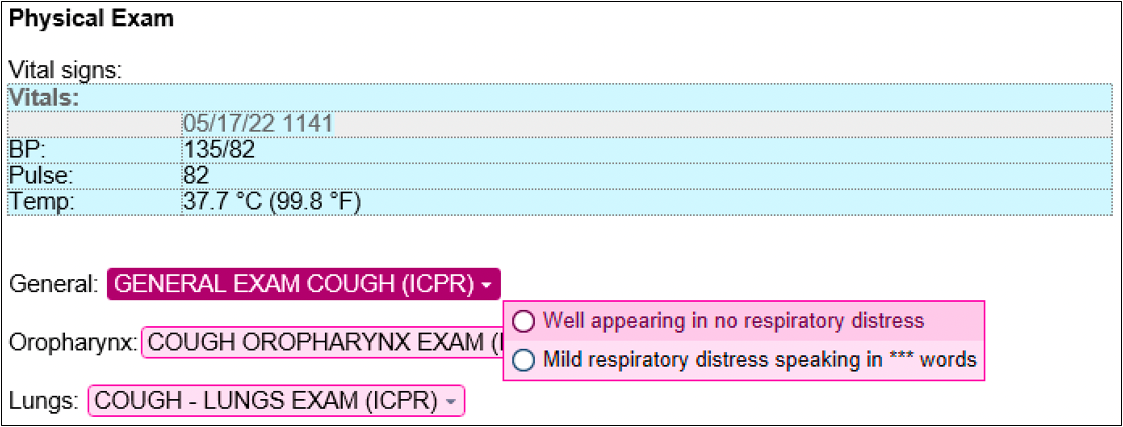

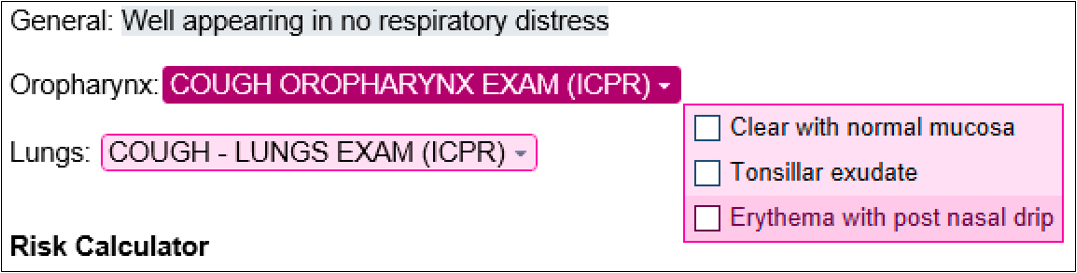

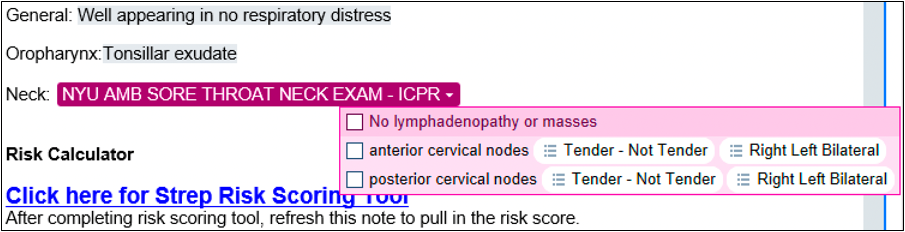
  
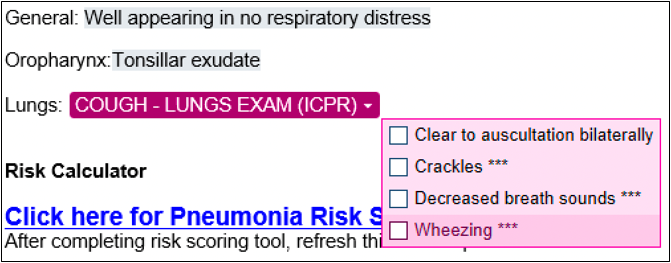


© 2022 Epic Systems Corporation
